# Assessment of Interruptive Behavior at Residency Teaching Conferences by Gender

**DOI:** 10.1001/jamanetworkopen.2020.33469

**Published:** 2021-01-15

**Authors:** Amrapali Maitra, Christina Langone, Olesya Baker, Patricia Foo, Julia Beamesderfer, Emily S. Lau, Maria A. Yialamas

**Affiliations:** 1 Department of Medicine, Brigham and Women’s Hospital, Boston, Massachusetts; 2 Department of Medicine, Highland Hospital–Alameda Health System, Oakland, California; 3 Department of Internal Medicine, Gallup Indian Medical Center, Gallup, New Mexico

## Abstract

This cohort study examines interruptions by residents and faculty discussants during residency teaching conferences by gender.

## Introduction

In anecdotal reports from residency retreats, female residents described a high frequency and negative impact of interruptive behavior by male colleagues.^1^ We sought to characterize interruptions during residency teaching conferences by gender.

## Methods

In this cohort study, we analyzed morning report conferences (1-hour case-based clinical reasoning sessions) in the Brigham and Women’s internal medicine residency program from January through June 2020. Because of precautions required during the coronavirus disease 2019 (COVID-19) pandemic, morning report conferences were held virtually (via Zoom [Zoom Video Communications]) beginning in March, allowing comparison of in-person and virtual conferences. Review and informed consent were not required by the Partners HealthCare institutional review board because the study was a quality improvement initiative. This study followed the Strengthening the Reporting of Observational Studies in Epidemiology (STROBE) reporting guideline.

A verbal interruption was defined as a breach in conversational turn-taking, including requests for clarification, agreement, disagreement, or change of subject.^2^ For consistency, a single trained team member (C.L.) recorded attendance and counted the number of interruptions at each conference. We classified interruptions according to the gender of the person being interrupted, the gender of the interrupter, and the roles of the interrupter and the person being interrupted (faculty interrupting resident, resident interrupting faculty, and so on). Gender was assigned based on external stereotypes.

Using Stata, version 16.1, (StataCorp LLC), we compared the median number of interruptions by men and women using a paired Wilcoxon signed rank test. We computed the expected number of interruptions by men by multiplying the proportion of men in a conference by the total number of interruptions of both male and female participants in that conference. Univariate descriptive statistics for the difference between observed and expected interruptions by men (baseline for no difference is 0) are reported and were compared by gender of the faculty discussant using a *t* test for equality of means. The *P* values were 2-sided, with statistical significance set at *P* = .05.

## Results

A total of 50 conferences (18 in-person and 32 virtual) were observed with a mean (SD) attendance of 22 (5.47) residents per conference. The mean (SD) gender distribution was 61.4% (13.8%) women and 38.6% (13.8%) men. In total, 187 interruptions were recorded: 126 (67%) by men and 61 (33%) by women. For men and women, the frequency of same-gender interruptions was not significantly different from that of opposite-gender interruptions.

The median number of interruptions by men was significantly higher than the median number of interruptions by women (1.5 [interquartile range {IQR}, 1.0-3.0] vs 0.5 [IQR, 0-2.0]; *P* = .007). The difference in the median number of interruptions by men and women was significant for virtual (1.0 [IQR, 0.5-2.0] vs 0 [IQR, 0-1.0]; *P* = .008) but not in-person (3.0 [IQR, 1.0-6.0] vs 1.5 [IQR, 0-1.4]; *P* = .13) conferences.

Across all conferences, men interrupted more often than expected based on attendance and assuming gender-equal participation (mean difference of observed vs expected, 0.97; 95% CI, 0.48-1.46). Interruptive behavior differed significantly by gender of the discussant across all conferences; men made fewer interruptions than expected with a female faculty discussant (mean difference, 0.17; 95% CI, −0.33 to 0.68) and more than expected with a male discussant (mean difference, 1.88; 95% CI, 1.10-2.67) (*P* = .001) (Table).

**Table.** Difference in Men’s Observed vs Expected Interruptions by Gender of the Faculty Discussant

Conference type	Faculty discussant		*P* value
	Female	Male	
All			
Conferences, No.	26	23	NA
Difference between observed vs expected interruptions, mean (95% CI)	0.17 (−0.33 to 0.68)	1.88 (1.10 to 2.67)	.001
In-person			
Conferences, No.	9	9	NA
Difference between observed vs expected interruptions, mean (95% CI)	−0.67 (−1.42 to 0.08)	2.88 (1.47 to 4.29)	<.001
Virtual			
Conferences, No.	17	14	NA
Difference between observed vs expected interruptions, mean (95% CI)	0.62 (0.41 to 1.37)	1.24 (0.35 to 2.13)	.45

Abbreviation: NA, not applicable.

## Discussion

The consequences of gender on communication and leadership in medical training is supported by an increasing literature.^3,4,5^ Our results suggest that men interrupt more frequently than women when adjusting for attendance. Men’s interruptive behavior was augmented by male faculty discussants.

In the communications literature, interruptions are perceived negatively as a mode of dominance because the interrupter violates the current speaker’s right.^6^ Observed differences in interruptive behavior are associated with various factors, including socialized gender roles around speaking and listening, perceived knowledge, and proportional representation (ie, male faculty reinforce communicative behaviors traditionally associated with men).^6^

Further research should include use of qualitative methods to explore the nature, tone, and content of interruptions and perceptions of the interrupted resident (possibly including loss of confidence or burnout). A limitation of this study was gender assignment by an observer rather than self-report. Larger sample sizes would allow for controlling of variables such as conference subject and postgraduate year level.

We found measurable gender differences in communication at conferences: male residents interrupted more frequently than female residents, consistent with “power and status effects.”^2^ Our findings highlight the need to foster gender equity in graduate medical education. Future interventions should include gender bias training around managing interruptions and greater inclusion of women faculty to diversify communication styles in the learning environment.
